# The Yellow Fever—Reward Offered

**Published:** 1884-06-20

**Authors:** 


### *THE YELLOW FEVER—REWARD OFFERED.*

Among the petitions which have been to the United States Senate "was one by Mr. Brown, from the Georgia Medical Society of Savannah and the Augusta Academy of Medicine, and the Board of Health of Augusta, praying that a reward may be offered by Congress for the discovery of the causes of the germ of yellow fever, with the mode of treatment that will prevent or cure the disease. In connection with this petition, Mr. Brown introduced a bill providing for the offer of a reward of $100,000 to any person who will discover the true cause or germ of yellow fever with any certain means of effecting its prevention, destruction or material modification, or who, without discovering the cause or germ of said disease, shall discover a certain and practical mode of effecting its prevention, destruction or material modification."

